# Sex Differences in the Association Between Poor Sleep Quality and Alcohol-Related Problems Among Heavy Drinkers With Insomnia

**DOI:** 10.3389/fnbeh.2022.875168

**Published:** 2022-05-19

**Authors:** Justin J. Verlinden, Mairead E. Moloney, Lauren N. Whitehurst, Jessica Weafer

**Affiliations:** ^1^Department of Psychology, University of Kentucky, Lexington, KY, United States; ^2^Department of Sociology, University of Kentucky, Lexington, KY, United States

**Keywords:** AUD, women, sleep disturbances, risky drinking, stress, AUDIT, PSQI

## Abstract

**Background:**

Alcohol Use Disorder (AUD) and insomnia are highly comorbid; at least 40% of individuals with AUD suffer from insomnia. Women are more likely to report insomnia than men and have seen a concerning rise in rates of AUD in recent years. As such, the association between AUD and insomnia could be particularly pronounced in women. However, currently little is known regarding sex differences in this association. Here we examined the degree to which relationships between alcohol use and sleep quality differ between women and men.

**Methods:**

Heavy drinking women (*n* = 66) and men (*n* = 45) completed the Pittsburgh Sleep Quality Index (PSQI) to assess sleep quality and the Alcohol Use Disorders Identification Test (AUDIT) to assess alcohol use and alcohol-related problems. Hierarchical regression analyses were conducted to determine sex differences in the association between poor sleep quality and alcohol-related problems.

**Results:**

After controlling for age, global subjective stress, and depression, sex significantly moderated the positive association between poor sleep quality and alcohol-related problems. Further analyses of the simple slopes for each sex revealed that poorer sleep quality (i.e., higher scores on the PSQI) were associated with greater alcohol-related problems (i.e., higher scores on the AUDIT) in women, but not in men.

**Conclusion:**

These results suggest that in heavy drinkers with insomnia, poor sleep is more strongly associated with drinking problems in women than in men. Future research is needed to investigate potential mechanisms underlying this relationship. Specifically, it will be important to determine whether sleep problems in heavy drinking women are a cause or consequence, or both, of heavy drinking.

## Introduction

At least 40% of individuals with alcohol use disorder (AUD) suffer from insomnia, suggesting these issues are highly comorbid (Zhabenko et al., 2012). This comorbidity is due in part to the bidirectional and feed-forward relationship between AUD and insomnia (Koob and Colrain, 2020). Excessive alcohol consumption, including both acute intoxication and withdrawal, negatively impacts sleep. Specifically, acute intoxication leads to more awakenings throughout the night, disruption of REM sleep, and reductions in sleep stages N1 and N2 (MacLean and Cairns, 1982; Williams et al., 1983; Chan et al., 2013). Additionally, alcohol withdrawal leads to insomnia, which can persist for several months following the cessation of drinking (Brower et al., 2011). Specifically, withdrawal is associated with decreases in slow wave sleep, N2 sleep, and REM latency and increases in N1 sleep and REM sleep, and these effects can persist for several months (Williams and Rundell, 1981; Drummond et al., 1998). Conversely, disrupted sleep contributes substantially to alcohol-related problems, in part because alcohol is often used as a sleep aid in an effort to self-medicate sleep disruptions (Johnson et al., 1998; Brower et al., 2001). Given this pronounced interconnection between insomnia and AUD, a better understanding of this relationship could improve intervention and prevention efforts for both disorders.

Associations between alcohol and sleep have historically been assessed in predominately male samples, with women underrepresented in these studies (Baker et al., 2020; Inkelis et al., 2020; Koob and Colrain, 2020). The lack of data on sleep-alcohol associations in women is especially concerning given that both insomnia and AUD are particularly problematic and increasingly prevalent in women. Specifically, women have a higher insomnia risk and self-report greater sleep disturbances and poorer sleep quality than men (Lindberg et al., 1997; Zhang and Wing, 2006; Zhang et al., 2016). Likewise, rates of AUD are increasing much more quickly among women compared to men (Grant et al., 2017). Such high rates of both insomnia and AUD in women highlight the need for studies to directly investigate the reciprocal influence of these disorders in women.

To date, a handful of studies have examined sex differences in associations between poor sleep and alcohol-related problems, and results have been mixed. In some studies, the negative association between sleep and drinking is stronger in men. For example, a large-scale longitudinal study found a bidirectional relationship between heavy drinking and sleeplessness in men, but not women (Rognmo et al., 2019). Additionally, Jaussent et al. (2011) reported that moderate alcohol use was associated with fewer insomnia symptoms in elderly women, whereas no relationship was observed in elderly men. By contrast, other studies have reported findings in the opposite direction, with a stronger negative association between sleep and alcohol use in women. For instance, Haario et al. (2013) found that insomnia was associated with higher rates of heavy drinking and binge drinking in women but not men. Further, in a sample of social alcohol and cannabis users, the association between risky alcohol use and poor sleep quality was stronger in women than men (Ogeil et al., 2015). Taken together, these findings suggest that among heavy drinkers specifically, women may experience stronger associations between alcohol and poor sleep quality than men.

It is important to note that the studies to date examining sex differences in associations between alcohol use and sleep have included predominately healthy, community-dwelling adults with relatively low levels of problem drinking and/or insomnia. Indeed, to our knowledge, no studies to date have examined sex differences in associations between alcohol and sleep in individuals meeting clinical criteria for both insomnia and problematic drinking. Given that alcohol-sleep associations are likely strengthened with increased levels of comorbid AUD and insomnia, this is an important gap in the literature. In order to address this gap, the current study examined sex differences in associations between problematic drinking and poor sleep quality in a sample of heavy drinkers with insomnia. Problem drinking was assessed with the Alcohol Use Disorders Identification Test (AUDIT; Babor et al., 1989) and poor sleep quality was assessed with the Pittsburgh Sleep Quality Index (PSQI; Buysse et al., 1989). We hypothesized that alcohol-related problems would be positively related to sleep disturbances (i.e., individuals with greater alcohol-related problems would also report poorer sleep quality). Moreover, we hypothesized that the association between poor sleep and alcohol problems would be more pronounced in women.

## Materials and Methods

### Participants

Male and female volunteers were recruited through online and printed advertisements for the parent study testing the efficacy of an insomnia intervention on sleep and drinking habits. The analyses presented here represent one of the aims of the larger project and were carried out with baseline data (collected prior to any intervention). Participants were required to be heavy drinkers [i.e., report weekly binge episodes (4/5 + drinks in one sitting for women/men) and an AUDIT score > 7] and to suffer from insomnia [i.e., self-report insomnia for three or more nights a week for the past 3 months and an Insomnia Severity Index (ISI) score > 14]. Additional inclusion criteria were: fluency in English, regular access to the internet, and 21–50 years old. Volunteers were excluded if they self-reported a previous diagnosis of AUD or substance use disorder, schizophrenia, bipolar disorder, or other psychotic spectrum disorder, sleep apnea, or if they were pregnant, lactating, or planning to become pregnant in the next 3 months.

### Procedure

The study was completed entirely online. Study data were collected and managed using REDCap electronic data capture tools hosted at the University of Kentucky (Harris et al., 2009, 2019). Eligible participants were emailed an invitation to the study along with a link to the consent form and baseline surveys. After electronically giving informed consent and agreeing to participate in the study by selecting yes on the online consent form, participants completed a battery of baseline surveys. Questionnaires assessed alcohol use, sleep quality, stress, and depression, and took approximately 30 min to complete. The Institutional Review Board of the University of Kentucky approved the study, and it was carried out in accordance with the Declaration of Helsinki. Upon completion of all baseline requirements for the parent study, participants were compensated $40 via Amazon gift card.

### Measures

Insomnia Severity Index (ISI; Bastien et al., 2001). This seven-item self-report questionnaire was included as a screening measure to assess eligibility based on severity of insomnia. Each item requires the subject to rate the severity of their symptoms on a Likert scale from 0 to 4. Scores range from 0 to 28 and are interpreted as follows: 0–7—no clinically significant insomnia; 8–14—subthreshold insomnia; 15–21—moderately severe clinical insomnia; 22–28—severe clinical insomnia.

Pittsburgh Sleep Quality Index (PSQI; Buysse et al., 1989). The PSQI is a 19-item self-report measure that provides a general index of sleep quality and sleep disturbances. It is composed of seven subscales assessing Sleep Quality, Sleep Latency, Sleep Duration, Sleep Efficiency, Sleep Disturbances, Use of Sleeping Medications, and Daytime Dysfunction. Each subscale is rated on a scale of 0–3 and then summed together, producing total scores ranging from 0 to 21. Scores of 6 or higher indicate poor sleep.

Alcohol Use Disorders Identification Test (AUDIT; Babor et al., 1989). The AUDIT was administered to assess alcohol-related problems. This is a 10-item self-report measure with scores ranging from 0 (no-alcohol related problems) to 40 (most severe alcohol-related problems). A score of 8 or higher typically indicates hazardous drinking.

Drinking Habits Questionnaire. Participants reported their typical number of drinking days and typical number of binge episodes (4/5+ drinks for women/men on one occasion) per month on the drinking habits questionnaire.

Perceived Stress Scale (PSS; Cohen et al., 1983). The PSS is a 10-item self-report measure used to gauge an individual’s global stress level by asking non-specific questions about how overloaded, uncontrollable, or unpredictable one’s life has been in the past month. All items are scored on a scale of 0 to 4, with higher scores indicating greater psychological distress.

Center for Epidemiologic Studies Depression Scale revised 10-item version (CESDR-10; Radloff, 1977). The CESDR-10 is a 10-item self-report measure that gauges a subject’s symptoms of depression. Each item is scored on a scale of 0 to 3, with higher scores indicating more severe symptoms.

### Analyses

Sex differences in all measures were analyzed by independent samples *t*-tests. We analyzed the degree to which impaired sleep was associated with alcohol-related problems by hierarchical regression analyses. Alcohol-related problems, as determined by AUDIT scores, served as the dependent variable. Age, PSS, and CESD-R scores were included as covariates and entered in Step 1. Age was included as a covariate due to marginal sex differences in our sample (see Table 1), and stress and depression scores were included as covariates due to well-established associations between these variables and both sleep [for reviews see Tsuno et al. (2005) and Kim and Dimsdale (2007)] and alcohol use [for reviews see Breese et al. (2011) and McHugh and Weiss (2019)]. Sex (male vs. female) and sleep quality, as determined by PSQI scores, were entered in Step 2. The interaction term (Sex × PSQI) was entered in Step 3 to determine whether the association between sleep quality and alcohol consumption differed by sex. Follow-up analyses tested the significance of the simple slopes for both men and women using the PROCESS extension tool (Hayes, 2017) in SPSS. Finally, bivariate correlations were conducted separately in men and women to assess associations between the individual PSQI subscales and AUDIT scores. We also include a qq plot (Supplementary Figure 1) justifying our choice of linear regression. No violations for linear regression were found, including non-normality of standardized residuals, form of relations, or heteroscedasticity.

**TABLE 1:** Mean age, drinking habits, depression, sleep quality, and stress in men and women.

	Women (*n* = 66)	Men (*n* = 45)	
	M (SD)	M (SD)	Contrasts
Age	24.8 (4.7)	26.8 (6.2)	ns
AUDIT	17.3 (7.2)	18.0 (7.0)	ns
Drinking (past 30 days)			
# of drinking days	15.0 (7.6)	18.0 (7.7)	Sig*
# of binge episodes	10.5 (5.8)	11.1 (6.8)	ns
CESD-R	16.9 (5.6)	14.9 (6.2)	ns
ISI	19.6 (3.1)	18.9 (2.8)	ns
PSQI	14.7 (2.5)	13.7 (2.8)	ns
PSS	24.6 (6.0)	22.6 (7.1)	ns

Group contrasts tested by independent samples t-tests; Sig* indicates p < 0.05.

## Results

### Sample Characteristics

A total of 111 participants (*n* = 45 male and *n* = 66 female) took part in this study. Sample characteristics are presented in Table 1. Men reported more drinking days in the past month compared to women (*p* = 0.04). No significant sex differences were observed on any other drinking or demographic measures (ps > 0.05). Both women and men had mean AUDIT scores well above the threshold for hazardous drinking, mean PSQI scores indicating poor sleep, and mean ISI scores that met criteria for moderately severe clinical insomnia. The racial makeup of the sample was White (*n* = 93), American Indian or Alaska Native (*n* = 3), Asian (*n* = 1), Black or African-American (*n* = 5), and Mixed Race (*n* = 9). The ethnic makeup of the sample was Non-Hispanic/Latino (n = 102), Hispanic/Latino (*n* = 7), and unreported (*n* = 2). Educational history was obtained from only a partial subset of the sample (*n* = 61, M = 15.43 years, SD = 1.89).

### Sex Differences in the Relationship Between Sleep Quality and Alcohol-Related Problems

**FIGURE 1:**
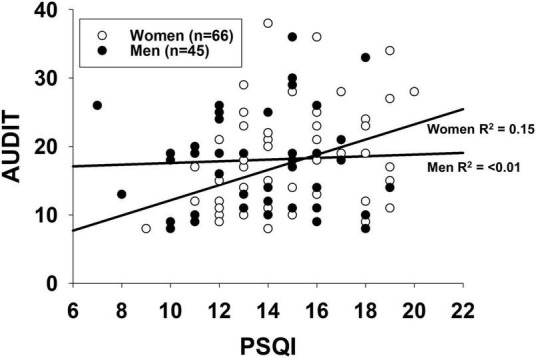
Bivariate correlations of Pittsburgh Sleep Quality Index (PSQI) and Alcohol Use Disorders Identification Test (AUDIT) scores in men and women. PSQI and AUDIT scores were significantly positively correlated in women (*p* < 0.01), but not in men (*p* = 0.75).

Results of the regression analysis testing sex differences in the association between sleep quality and alcohol problems are presented in Table 2. Of the three covariates included in the model, only the PSS was significantly related to AUDIT scores and this effect was seen in all three steps of the model. This effect suggested that individuals with more stress had higher AUDIT scores. PSQI scores were also positively related to AUDIT scores in the second and third steps of the model, such that individuals with poorer sleep quality reported greater alcohol-related problems. Moreover, the Sex × PSQI interaction term was significant (*p* = 0.04), indicating that the association between PSQI and AUDIT scores differed by sex. Using the PROCESS extension tool (Hayes, 2017) in SPSS, we probed this interaction by testing the significance of the simple slopes for both women and men. Women showed a significant, positive relationship between PSQI and AUDIT scores, B = 1.09, *t* = 3.20, *p* < 0.01. By contrast, PSQI scores were not significantly related to AUDIT scores in men, *B* = 0.04, *t* = 0.10, *p* = 0.92. Figure 1 shows the bivariate relationship between PSQI and AUDIT scores for women and men separately.

**TABLE 2:** Hierarchical regression analysis of sex differences in the relationship between sleep quality and alcohol-related problems.

AUDIT scores			
	
	R^2^	B (se)	Beta
**Step 1**	0.06		
Age		0.03 (0.12)	0.02
PSS		**0.32 (0.15)**	**0.29** *
CESD-R		−0.09 (0.17)	–0.07
**Step 2**	**0**.11*		
Age		−0.05 (0.13)	–0.04
PSS		**0.32 (0.15)**	0.30*
CESD-R		−0.15 (0.17)	–0.13
Sex		1.74 (1.38)	0.12
PSQI		**0.63 (0.26)**	0.24*
**Step 3**	0.15*		
Age		−0.01 (0.13)	–0.01
PSS		**0.34 (0.15)**	0.31*
CESD-R		−0.14 (0.16)	–0.12
Sex		1.57 (1.36)	0.11
PSQI		**1.09 (0.34)**	0.41**
Sex × PSQI		−**1.05 (0.50)**	−0.26*

*p < 0.05, **p ≤ 0.01. Bold values indicate p < 0.05.

### Bivariate Correlations Between Pittsburgh Sleep Quality Index Subscales and Alcohol Use Disorders Identification Test Scores in Men and Women

Bivariate correlations between each of the PSQI subscales and AUDIT scores were conducted separately in men and women. Results of these correlations can be found in Table 3. Two significant associations were seen in women. Sleep Duration, *r* = 0.40, *p* = 0.001, and Sleep Disturbances, *r* = 0.28, *p* = 0.02, were significantly positively associated with AUDIT scores such that less total sleep time and more sleep disturbances were associated with greater AUDIT scores. By contrast, there were no significant associations between any of the subscales and AUDIT scores in men.

**TABLE 3:** Pearson correlations (r) between individual Pittsburgh Sleep Quality Index (PSQI) subscales and Alcohol Use Disorders Identification Test (AUDIT) scores in men and women.

	Women (*n* = 66)	Men (*n* = 45)
	
	AUDIT	AUDIT
Subjective sleep quality	0.23	0.27
Sleep latency	0.01	−0.07
Sleep duration	**0**.40**	−0.11
Habitual sleep efficiency	0.15	0.27
Sleep disturbances	0.28*	0.20
Use of sleep medication	0.12	−0.13
Daytime drowsiness	0.10	0.01

*p < 0.05, **p ≤ 0.01. Bold values indicate p < 0.05.

## Discussion

This study examined sex differences in associations between sleep quality and alcohol use in a sample of heavy drinkers with insomnia. Findings showed that poor sleep quality was significantly associated with more alcohol-related problems in women, but not in men. This effect was found even after controlling for several potential confounding variables such as age, global stress, and symptoms of depression. Based on bivariate correlational analyses, the PSQI Sleep Duration and Sleep Disturbances subscales may be driving the observed relationship. In line with previous research (Rice and Van Arsdale, 2010; Tavolacci et al., 2013; Ruisoto et al., 2020), we also found that stress was associated with alcohol-related problems. These findings build on initial studies which have suggested a stronger relationship between sleep and alcohol use in women compared to men (Haario et al., 2013; Ogeil et al., 2015), and help clarify some of the mixed findings from previous reports.

This is the first study, to our knowledge, to test sex differences in the association between poor sleep and alcohol-related problems in a sample of heavy drinkers with insomnia. Prior reports have come primarily from community samples with relatively low rates of problematic drinking, and these studies have typically shown a stronger association between poor sleep and alcohol-related problems in men. The one exception is the study conducted by Ogeil et al. (2015) that tested sex differences in sleep-alcohol associations among social drinkers (i.e., mean AUDIT scores = 8/11 for women/men). This study found that among the riskiest drinkers, sleep and alcohol were more strongly linked in women than men. Our results take this a step further and show that among heavy drinkers at very high risk (i.e., mean AUDIT scores = 17/18 for women/men), the association is especially pronounced in women and non-existent in men. Taken together, these findings suggest that poor sleep is especially likely to accompany heavy drinking in women.

The sex difference in the association between poor sleep and alcohol-related problems observed here lays the groundwork for future studies investigating sex differences in the directionality of this relationship. One hypothesis is that disrupted sleep is more likely to lead to increased alcohol consumption in women than in men. While there is clear evidence from prospective studies to suggest that insomnia and sleep disturbances can play a causal role in the onset of alcohol use and alcohol-related problems (Wong et al., 2004, 2015; Roberts et al., 2008; Hasler et al., 2014, 2016), little is known about sex differences in this relationship. Additionally, a significant number of drinkers report using alcohol to self-medicate insomnia (Johnson et al., 1998; Brower et al., 2001), in which case poorer sleep would lead to greater alcohol use. Although an initial report suggested such self-medication may be more common in men (Johnson et al., 1998), it is possible that this trend may be changing with increasing alcohol use among women. In sum, future studies are needed to determine whether the current findings suggest that poor sleep in women is playing a causal role in increased alcohol consumption.

Alternatively, another hypothesis suggests that greater alcohol use leads to poorer sleep for women than for men (i.e., women may be more sensitive than men to the acute impairing effects of alcohol on sleep). Indeed, one laboratory study (Arnedt et al., 2011) suggests that this is the case. In this study, young adult social drinkers consumed an oral dose of alcohol (target breath alcohol concentration = 100 mg %) 1 h prior to bedtime. Sleep was monitored throughout the night using polysomnography, and results showed that alcohol impaired objective sleep quality indicators to a greater extent in women than in men. Specifically, alcohol decreased total sleep time and sleep efficiency, and increased number of awakenings and minutes awake after sleep onset, in women, but not in men. These findings suggest that our observed association between poor sleep and alcohol use in women may be because heavy drinking causes more pronounced sleep impairment in women.

This study has some limitations. First, there were more women than men in our sample. The observed effect size in men for the relationship between sleep and drinking was quite low, suggesting that the sex difference was not due to greater power in women than in men. However, it will be important to replicate these findings with equal numbers of each sex. Additionally, study measures consisted entirely of self-report measures. Future studies would benefit from inclusion of more objective measures, including actigraphy watches and polysomnography to assess sleep and wrist biosensors to assess alcohol consumption.

In sum, our findings offer important contributions to the literature. Our primary finding, that poor sleep is linked to greater alcohol problems in heavy drinking females (but not males) with insomnia, is particularly novel. These findings further support the hypothesis that sleep quality and alcohol use are linked in women who drink heavily. Future studies are needed to determine the degree to which poor sleep is a sex-specific cause or consequence (or both) of heavy drinking in women. These studies may benefit in focusing specifically on the role of sleep duration and sleep disruptions, since our results point to those two factors as being particularly associated with alcohol problems in women but not in men. This line of research has the potential to contribute important information for the development of sex-specific prevention and treatment efforts for female heavy drinkers, particularly women who suffer from both alcohol-related problems and insomnia.

## Data Availability Statement

The raw data supporting the conclusions of this article will be made available by the authors, without undue reservation.

## Ethics Statement

The studies involving human participants were reviewed and approved by University of Kentucky Institutional Review Board. The patients/participants provided their written informed consent to participate in this study.

## Author Contributions

JW and MM designed the study. JV collected the data, conducted the statistical analyses, and wrote the first draft of the manuscript. All authors revised the manuscript critically for intellectual content and approved the final version.

## Conflict of Interest

The authors declare that the research was conducted in the absence of any commercial or financial relationships that could be construed as a potential conflict of interest.

## Publisher’s Note

All claims expressed in this article are solely those of the authors and do not necessarily represent those of their affiliated organizations, or those of the publisher, the editors and the reviewers. Any product that may be evaluated in this article, or claim that may be made by its manufacturer, is not guaranteed or endorsed by the publisher.
