# Supplementary figures and images for: Sex Differences in the Association Between Poor Sleep Quality and Alcohol-Related Problems Among Heavy Drinkers With Insomnia
Source: Front Behav Neurosci. 2022 May 19;16:875168. doi: 10.3389/fnbeh.2022.875168 (PMC9161212; doi:10.3389/fnbeh.2022.875168)

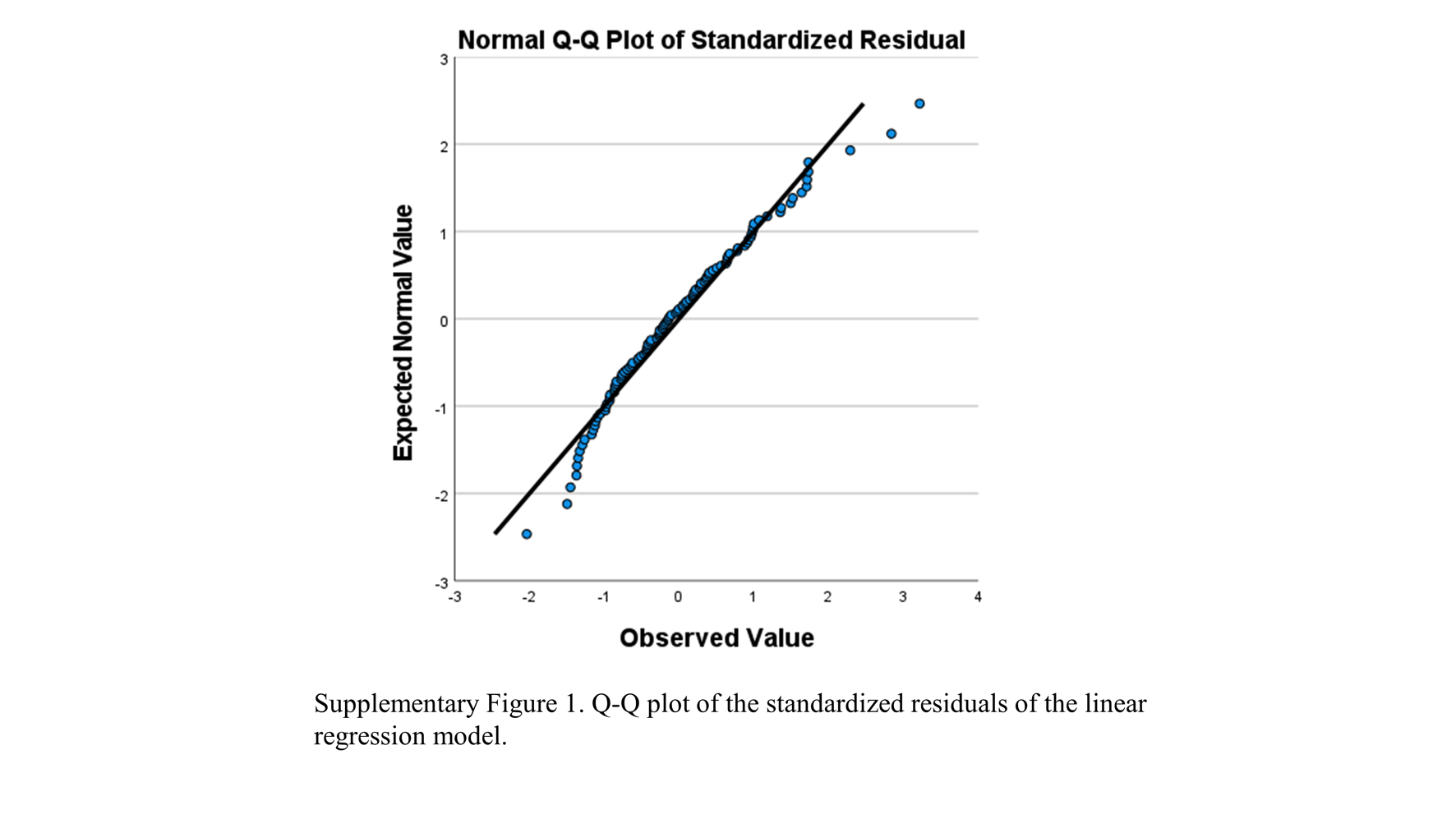

Supplement: Supplementary file 1 [file Image_1.tif]
